# Increased Central and Peripheral Thyroid Resistance Indices During the First Half of Gestation Were Associated With Lowered Risk of Gestational Diabetes—Analyses Based on Huizhou Birth Cohort in South China

**DOI:** 10.3389/fendo.2022.806256

**Published:** 2022-03-08

**Authors:** Zhao-min Liu, Guoyi Li, Yi Wu, Di Zhang, Sujuan Zhang, Yuan-Tao Hao, Weiqing Chen, Qi Huang, Shuyi Li, Yaojie Xie, Mingtong Ye, Chun He, Ping Chen, Wenjing Pan

**Affiliations:** ^1^ Guangdong Provincial Key Laboratory of Food, Nutrition and Health, School of Public Health, Sun Yat-sen University, Guangzhou, China; ^2^ School of Public Health, Sun Yat-sen University (North Campus), Guangzhou, China; ^3^ School of Nursing, Hong Kong Polytechnic University, Hong Kong, Hong Kong SAR, China; ^4^ Huizhou First Mother and Child Health-Care Hospital, Huizhou, China

**Keywords:** thyroid function tests, thyroid hormone resistance index, gestational diabetes mellitus, prevention, thyroid

## Abstract

**Objectives:**

The study aimed to explore the relationship of thyroid function and resistance indices with subsequent risk of gestational diabetes (GDM).

**Design:**

This was a longitudinal study embedded in the Huizhou Birth Cohort.

**Methods:**

A total of 2,927 women of singleton pregnancy were recruited from January to October of 2019. Thyroid central resistance indices were evaluated by Thyroid Feedback Quartile-Based index (TFQI), Thyrotrophy T4 Resistance Index (TT4RI), and TSH Index (TSHI) based on plasma-free thyroxine (FT4) and thyroid-stimulating hormone (TSH) levels during the first half of pregnancy. Thyroid peripheral sensitivity was assessed by free triiodothyronine (FT3) to FT4 ratio (FT3/FT4), a proxy of deiodinase activity. GDM was diagnosed between 24 and 28 weeks of gestation by a standardized 75 g oral glucose tolerance test. Multivariable linear and logistic regression was applied to examine the associations of thyroid markers with GDM risk.

**Results:**

FT3 and FT3/FT4 were positively associated with both fasting and post-load glucose levels, while TSH, TSHI, TT4RI, and TFQI were negatively associated with 1 and 2 h post-load glucose levels. Compared with the lowest quartile, GDM risk in the highest quartile increased by 44% [odds ratio (OR) = 1.44; 95%CI, 1.08–1.92; *p_trend_* = 0.027] for FT3 and 81% (OR = 1.81; 95%CI, 1.33–2.46; *p_trend_* < 0.001) for FT3/FT4, while it lowered by 37% (OR = 0.63; 95%CI, 0.47–0.86; *p_trend_* = 0.002] for TSHI, 28% for TT4RI (OR = 0.72; 95%CI, 0.54–0.97; *p_trend_* = 0.06), and 37% for TFQI (OR = 0.63; 95%CI, 0.46–0.85; *p_trend_* < 0.001).

**Conclusions:**

This longitudinal study indicated that higher FT3 and FT3/FT4 and lower central thyroid resistance indices were associated with increased risk of GDM.

## Introduction

Pregnancy has a considerable impact on thyroid function. Thyroid gland enlarges nearly by 40%, and its hormone production increases by 50% during gestation (1). In early pregnancy, increased placental human chorionic gonadotropin (HCG) stimulates thyroid hormones [THs, including thyroxine (T4) and triiodothyronine (T3)] secretion and causes a reduction in thyroid-stimulating hormone (TSH) (2, 3). Thyroid diseases are the second most common endocrine disorders affecting women of reproductive age (4). About 10%–15% of pregnant women suffer from thyroid disorders during pregnancy including overt and subclinical hyper- or hypothyroidism (2, 5). Maternal thyroid disorders have been often associated with adverse obstetric complications and outcomes (2, 6). However, the utility of thyroid function screening during early pregnancy is still controversial, and its implementation varied from country to country (2).

As the major anabolic hormones, THs are critical regulators of energy metabolism and glucose homeostasis (7), and even variations within normal range might result in significant metabolic consequences (8). Gestational diabetes mellitus (GDM) is the most prevalent disease among pregnant women, affecting up to 15%–25% of pregnancies worldwide (9) and a recent report of 22.9% in Guangdong, South China (10). Subclinical thyroid disorders and GDM occur frequently during pregnancy (2). Several meta-analyses have suggested that women of subclinical hypothyroidism [SCH, defined as increased TSH while normal free T4 (FT4)] had increased risk of GDM (11, 12) with greater risk being observed in early pregnancy than the later stages (11, 13). However, longitudinal studies on the associations of thyroid markers with GDM risk were limited and inconclusive. Differences in study design, ethnics, availability of laboratory data (i.e., thyroid antibodies), different cutoffs for TSH, and diagnosis criteria for GDM might contribute to the discrepant findings.

Owing to an inverse feedback loop, TSH and THs are physiologically and inversely correlated. T3 and T4 inhibit TSH secretion and synthesis and decrease the sensitivity of the thyrotrophs to TSH. However, recent evidence showed that the co-existence of high T4 and high TSH in diabetic patients may implicate impaired thyroid sensitivity, a syndrome of reduced responsiveness of target tissues to THs (14). Thyroid sensitivity or resistance and their relationship with metabolic disorders received increasing attention in recent years (14–17), suggesting an additional cardio-metabolic trait predicting metabolic disorders (18). Resistance to thyroid hormone (RTH) can be systematized into central resistance, which affects the feedback set point in the pituitary, and peripheral resistance, which decreases the metabolic effects of THs on tissues (19). RTH, evaluated by circulating FT4 and TSH levels, predominantly reflects central or pituitary resistance, the grade of pituitary gland inhibition by FT4 levels (19). Free T3 (FT3) is converted from FT4 by deiodinase in peripheral tissues; thus, an FT3 to FT4 ratio (FT3/FT4) could be served as not only a proxy of deiodinase activity but also the peripheral sensitivity to THs (20). Compared with a single thyroid marker, the composite indices could systematically reflect the regulation of thyroid hormone homeostasis (21). It is uncertain whether TH insensitivity impacts the development of GDM. Exploration of such an association during gestation would have essential clinical significance, as thyroid hormones change dramatically since pregnancy. We thus based on an ongoing birth cohort to explore the temporal associations of THs and thyroid resistance indices with subsequent risk of GDM in an iodine-sufficient area of South China.

## Materials and Methods

This was a longitudinal study conducted in a tertiary mother and child hospital in Huizhou city, Guangdong province, South China. Eligible singleton pregnant women of 18–49 years were enrolled between 10 and 20 weeks of gestation during their first antenatal visit in the outpatient clinic for nutrition consultation from January to October 2019. Exclusion criteria were women of pre-existing diabetes or thyroid disorders or undergoing medication treatment for diabetes or thyroid dysfunction and unavailable results of thyroid testing before 20 weeks or oral glucose tolerance test (OGTT) before 28 weeks of gestation. Ethical approval has been obtained from the Ethical Research Committee of Huizhou First Mother and Child Health-Care Hospital. All participants provided written informed consents before enrolment.

Individual information was collected by trained research staff *via* face-to-face interview using a pretested questionnaire including socio-demographics, family and medical history, medication usages, dietary habits, and other lifestyle factors (i.e., physical activity, smoking, and alcohol drinking) in pre- and early pregnancy. All the biochemical data were retrieved from the Hospital Information Management System. Maternal blood samples were collected after 8–12 h overnight fasting and centrifuged for 10 min at 3,000 rpm. Thyroid markers including plasma FT3, FT4, and TSH (Roche Diagnostics, Indianapolis, IN, USA) were quantified by immunochemiluminometric assay (ICMA) on the Roche Cobas Elesys 602 Analyzer in the certified clinical laboratory of the hospital. Thyroid antibodies (TAs) including antibodies of thyroperoxidase (TPOAb), TSH receptor (TRAb), and anti-thyroglobulin (TGAb) were measured only for those with abnormal levels of THs (FT3 <3.1 or >6.8 pmol/L, FT4 <12 or >22 pmol/L, TSH <0.27 or >4.2 mIU/L, according to the reference ranges of commercial reagent kits) by immunoassay. Antibodies were regarded as positive when >34 IU/L for TPOAb, 1.75 IU/L for TRAb, and 115 IU/L for TGAb. All the intra- and inter-assay coefficients of variation for thyroid biomarkers were <10%. Central thyroid resistance indices were evaluated by Thyrotroph T4 Resistance Index [TT4RI, TT4RI = FT4 (pmol/L) × TSH (mIU/L)], TSH index [TSHI, TSHI = ln TSH (mIU/L) + 0.1345 × FT4 (pmol/L)], and Thyroid Feedback Quartile-Based index [TFQI, TFQI = cdf FT4 − (1 − cdf TSH); cdf: cumulative distribution function] based on circulating FT4 and TSH levels using established formula (18, 19). TSHI defines the maximum possible TSH response in the FT4-uninhibited state at a theoretical FT4 value of 0. TFQI indicated the difference between FT4 quantile and the reversed TSH quantile based on the empirical joint distribution of FT4 and TSH. Ranks of FT4 and TSH were converted to quantiles between 0 and 1, taking into account the sampling weights. The positive values suggested that the HPT axis is less sensitive to the change in FT4. The peripheral thyroid sensitivity was assessed by FT3/FT4, a proxy of deiodinase activity.

In order to improve the accuracy and validity of diagnosis of thyroid disorders (2), we have established the trimester- and assay-specific reference intervals (2.5th–97.5th percentile) for FT4 and TSH among a subsample of women (n = 602) who were all TPOAb negative (**Supplementary Table S1**). Overt and subclinical thyroid dysfunctions were determined in accordance with the newly established reference intervals. All pregnant women underwent a 2-h of 75 g OGTT during 24–28 gestational weeks as part of their routine antenatal screening for GDM. GDM was diagnosed according to the recommendation of International Association of Diabetes and Pregnancy Study Groups (IADPSG) if either of fasting or 1 or 2 h post-load glucose level above their respective cutoffs: 0 h ≥ 5.1 mmol/L, 1 h ≥ 10.0 mmol/L, or 2 h ≥ 8.5 mmol/L (22).

## Statistical Analysis

Statistical analysis was conducted using SPSS 21.0 software. Participants’ characteristics and selected risk factors were compared by GDM and non-GDM groups using either Student’s t-test or chi-square test. Data distribution and variance heterogeneity were tested before comparisons. Multivariable linear regression was conducted to examine the associations of thyroid variables with plasma glucose levels at fasting and 1 and 2 h post-load. Multivariable logistic regression was applied to estimate the crude and full-adjusted odds ratios (ORs) of GDM by quartiles of THs (FT3, FT4, and TSH) and thyroid peripheral sensitivity (FT3/FT4) and central resistance indices (TT4RI, TSHI, and TFQI), respectively. Tests of linear trend were performed by using the median of each quartile as a continuous variable in the regression models. Potential confounders were chosen based on the literature and results of univariate or biological potentials, which included maternal age (years), education, body mass index (BMI) of pre-pregnancy (kg/m^2^), multi-parity (yes or no), smoking (yes or no) and alcohol drinking during early pregnancy (yes or no), and family history of diabetes (yes or no). Thyroid variables were further standardized to estimate GDM risk with one standard deviation (SD) changes. Thyroid disorders during gestation including subclinical and clinical hyper- and hypothyroidism and isolated hypothyroxinemia (IH) were determined according to the newly established reference intervals. GDM risk (ORs) was estimated by logistic regression analysis for patients of various thyroid dysfunction with women of euthyroid as the reference group. Sensitivity analyses were conducted among women of euthyroid status according to the newly established reference intervals or by exclusion of those of any positive thyroid antibodies. The characteristics of women who were included for analysis (n = 2,927) were compared with women who were excluded (n = 644) due to unavailable OGTT results or thyroid testing later than 20 gestational weeks. Subgroup analyses were performed to testify the results’ consistency by stratification of maternal age (≤35 vs. >35 years), body mass index (BMI, <24.0 vs. ≥24.0 kg/m^2^) (23), parity (null vs. ≥1), and family history of diabetes (yes vs. no), as they were the established risk factors for GDM. Interactions were tested before subgroup analyses, and results were only reported for p for interaction <0.15.

## Results

**Figure 1:**
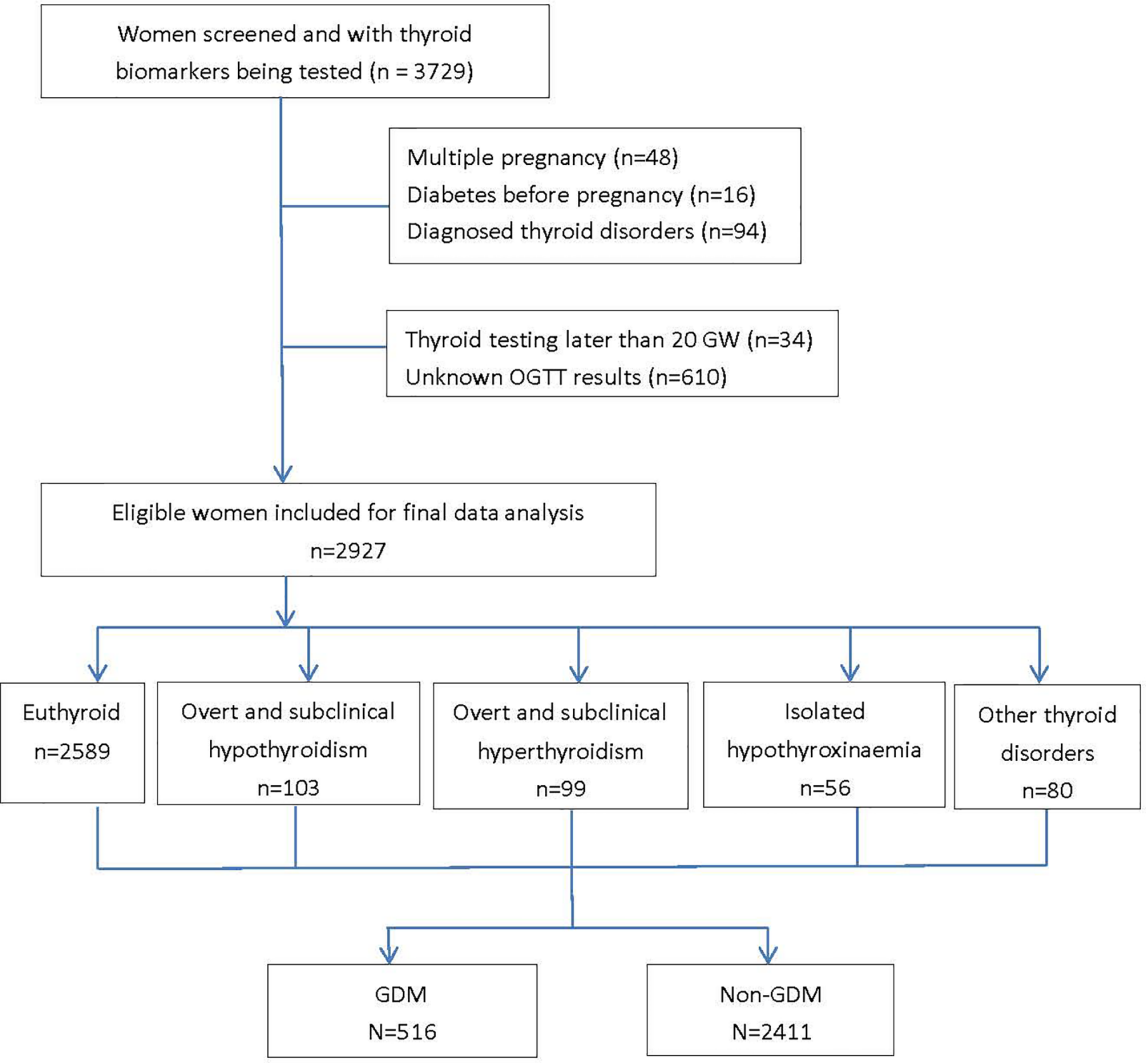
Based on the reference ranges of thyroid hormones established based on local population, the prevalence of thyroid disorders during the first half of pregnancy were 3.4% (n=100) for subclinical hypothyroidism, 0.1% (n=3) for overt hypothyroidism, 0.8% (n=23) for subclinical hyperthyroidism, 2.6% (n=76) for overt hyperthyroidism and 1.9% (n=56) for isolated hypothyroxinaemia (IH) in our participants. The diagnoses of overt and subclinical hyper- and hypo- hyperthyroidism were determined based on the newly established reference intervals of FT4 and TSH which were population and trimester specific. GDM, gestational diabetes mellitus; GW, gestational weeks; OGTT, oral glucose tolerance test.

A total of 3,729 were screened and had completed thyroid data, among which 2,927 women were included for analyses (response rate, 78.5%) with an average age of 28.3 ± 4.4 years, pre-pregnancy BMI of 21.2 ± 3.5 kg/m^2^, and 51.0% of primi-parity. A total 516 women (17.6%) developed GDM in the second trimester (**Figure 1**, study flow chart). Compared with the non-GDM group, GDM patients were more likely to have a higher rate of family history of diabetes, higher pre-pregnancy BMI, and plasma FT3 and FT3/FT4 levels (**Table 1**). Results of multivariable linear regression (**Table 2**) indicated that FT3 and FT3/FT4 were positively associated with both fasting and post-load glucose levels with the standardized coefficients (β) ranging from 0.038 to 0.107 (all *p* < 0.05 except a marginal significance of 0.054 between FT3/FT4 and fasting glucose level), while TSH, TSHI, TT4RI, and TFQI were negatively associated with 1 and 2 h post-load glucose levels with β of −0.067 to −0.034 (all *p* < 0.05 except a marginal significance of *p* =0.056 between TSHI and 1 h glucose level). The assumption of linear regression has been tested by residual plot, which indicated that the dots of residuals were randomly dispersed around the x-axis. Compared with the lowest quartile of FT3 and FT3/FT4, women in the highest quartile group had significantly increased risk of GDM by 44% (OR, 1.44; 95% CI, 1.08–1.92; *p_trend_* = 0.027) for FT3 and 81.0% (OR, 1.81; 95% CI, 1.33–2.46; *p_trend_* < 0.001) FT3/FT4 (**Table 3**). FT4 was, in general, inversely associated with GDM risk but with the lowest risk being observed in Q2 group (OR, 0.69; 95% CI, 0.53–0.92). Women with 1 SD increase in FT3 and FT3/FT4 were significantly associated with increased risk of GDM by 12% (OR, 1.12; 95% CI, 1.01–1.24) and 31% (OR, 1.31; 95% CI, 1.18–1.46), respectively. One SD increase in TSH was associated with decreased GDM risk by 11% (OR, 0.89; 95% CI, 0.80–0.99). For the associations of central thyroid resistance indices with GDM risk (**Table 3**), women in the highest quartile were significantly associated with reduced risk of GDM by 37% for TSHI (OR, 0.63; 95%CI, 0.47–0.86, *p_trend_* = 0.002), 28% for TT4RI (OR, 0.72; 95%CI, 0.54–0.97, *p_trend_* = 0.026), and 37% for TFQI (OR, 0.63; 95%CI, 0.46–0.85, *p_trend_* = 0.001) in comparison with the lowest quartile. One SD increase in TT4RI and TFQI were associated with decreased GDM risk by 15% and 18%, respectively.

**Table 1:** Participants’ characteristics and selected risk factors by gestational diabetes status during the first trimester (n = 2,927).

	Non-GDM (N = 2,411)	GDM (N = 516)	*p* value
Maternal age (years)	28.0 ± 4.1	29.8 ± 4.3	<0.001
Pre-pregnancy BMI (kg/m^2^)	20.9 ± 3.2	22.6 ± 4.1	<0.001
Education, n (%)			0.242
Below high school	502 (20.8)	112 (21.7)	
High school or equivalent	601 (24.9)	129 (25.0)	
College/university and above	1,308 (54.2)	275 (53.3)	
Multi-parity, n(%)	1,244 (51.6)	249 (48.3)	0.061
Family history of type 2 diabetes, n(%)	172 (7.1)	67 (13.0)	<0.001
Smoking during early pregnancy, n(%)	25 (1.1)	8 (1.6)	0.090
Alcohol drinking during early pregnancy, n(%)	93 (3.9)	17 (3.3)	0.554
FT3 (pg/ml)	4.94 ± 1.20	5.08 ± 1.68	0.033
FT4 (pmol/L)	17.26 ± 3.66	16.74 ± 4.66	0.006
TSH (mIU/L)	1.48 ± 2.00	1.33 ± 1.06	0.104
FT3/FT4	0.29 ± 0.04	0.31 ± 0.05	<0.001

For continuous variables, data are presented as mean ± standard deviation and compared by Student’s t-test. For categorical variables, data are presented as n (%) and compared by chi-square test. Smoking was defined as smoking at least once during the first trimester; alcohol drinking was defined as alcohol drinking at least once per week with more than 100 ml per time during the 1st trimester. GDM, gestational diabetes mellitus; BMI, body mass index; FT4, free thyroxine; FT3, free triiodothyronine; TSH, thyroid-stimulating hormones.

**Table 2:** Multivariable linear regression on the associations of thyroid markers with levels of fasting and post-load glucose by 75 g oral glucose tolerance test (OGTT).

	Fasting glucose (mmol/L)		1 h glucose level of OGTT (mmol/L)		2 h glucose level of OGTT (mmol/L)	
	β (95%CI)	*p*	β (95%CI)	*p*	β (95%CI)	*p*
**FT3** (pg/ml)	0.040 (0.003, 0.078)	0.032	0.082 (0.047, 0.119)	<0.001	0.075 (0.040, 0.111)	<0.001
**FT4** (ng/dl)	0.012 (−0.025, 0.050)	0.517	−0.001 (−0.038, 0.035)	0.937	−0.005 (−0.041, 0.031)	0.796
**TSH** (mIU/L)	−0.010 (−0.046, 0.026)	0.589	−0.058 (−0.093, 0.023)	0.001	−0.059 (−0.093, 0.024)	0.001
**FT3/FT4**	0.038 (−0.001, 0.077)	0.054	0.105 (0.068, 0.143)	<0.001	0.107 (0.070, 0.144)	<0.001
**TSHI**	−0.018 (−0.054, 0.018)	0.328	−0.034 (−0.070, 0.001)	0.056	−0.060 (−0.089, 0.019)	0.001
**TT4RI**	−0.014 (−0.051, 0.022)	0.432	−0.064 (−0.099, 0.028)	<0.001	−0.054 (−0.102, 0.032)	0.002
**TFQI**	−0.015 (−0.052, 0.022)	0.435	−0.043 (−0.079, 0.007)	0.020	−0.067 (−0.091, 0.019)	<0.001

Multivariable linear regression was applied with adjustment being made for maternal age (year), education, smoking (yes or no) and alcohol drinking during early pregnancy (yes or no), BMI of pre-pregnancy (kg/m^2^), multi-parity (yes or no), and family history of diabetes (yes or no). β, standardized coefficients; FT3, free triiodothyronine; FT4, free thyroxine; TSH, thyroid-stimulating hormone; TSHI, TSH Index; TT4RI, Thyrotroph T4 Resistance Index. TT4RI = FT4 (pmol/L) × TSH (mIU/L); TSHI = ln TSH (mIU/L) + 0.1345 × FT4 (pmol/L); TFQI = cdf FT4 − (1 − cdf TSH). TFQI indicated the difference between FT4 quantile and the reversed TSH quantile; Cdf denoted cumulative distribution function.

**Table 3:** Odds ratios (ORs) and 95% confidence intervals (CIs) of gestational diabetes by quartiles and per SD increment of thyroid function markers and peripheral and central thyroid resistance indices during first trimester among 2,927 pregnant women.

Thyroid hormones (min–max)	GDM n (%)	Crude OR (95%CI)	Adjusted OR (95%CI)	Per SD OR (95%CI)
**Thyroid function markers**				
** FT3 (pg/ml)**				1.12 (1.01, 1.24)
** **Q1(2.84–)	115(15.4)	1.00	1.00	
** **Q2(4.45–)	119(16.5)	1.09(0.82, 1.44)	1.08 (0.80, 1.45)	
** **Q3(4.85–)	121(16.5)	1.09(0.82, 1.44)	0.98 (0.73, 1.32)	
** **Q4(5.28–10.75)	161(22.2)	1.57(1.21, 2.05)	1.44 (1.08, 1.92)	
** **P for trend		0.001	0.027	
** FT4 (pmol/L)**				0.90 (0.80, 1.01)
** **Q1(8.62–)	174(23.8)	1	1	
** **Q2(15.29–)	119(16.2)	0.62(0.48, 0.80)	0.69(0.53, 0.92)	
** **Q3(16.67–)	115(15.8)	0.60(0.46, 0.78)	0.76(0.57, 0.996)	
** **Q4(18.26–48.51)	108(14.8)	0.56(0.43, 0.73)	0.78(0.59, 1.04)	
** **p for trend		<0.001	0.109	
** TSH (mIU/L)**				0.89 (0.80, 0.99)
** **Q1(0.0025–)	131(17.9)	1	1	
** **Q2(0.61–)	140(19.1)	1.08(0.83, 1.41)	1.04(0.79, 1.37)	
** **Q3(1.21–)	135(18.4)	1.04(0.80, 1.35)	0.99(0.75, 1.31)	
** **Q4(1.98–7.93)	110(15.1)	0.81(0.62, 1.07)	0.78(0.58, 1.04)	
** **p for trend		0.148	0.087	
**Peripheral thyroid resistance index**				
** FT3/FT4**				1.31 (1.18, 1.46)
** **Q1(0.10–)	86(11.7)	1	1	
** **Q2(0.26–)	114(15.6)	1.39(1.03, 1.87)	1.28(0.94, 1.76)	
** **Q3(0.29–)	133(18.2)	1.67(1.25, 2.24)	1.39(1.02, 1.90)	
** **Q4(0.32–0.64)	183(25.0)	2.50(1.89, 3.31)	1.81(1.33, 2.46)	
** **p for trend		<0.001	<0.001	
**Central thyroid resistance indices**				
** TSHI**				0.91 (0.83, 1.01)
** **Q1(−4.04–)	141(19.3)	1	1	
** **Q2(1.74–)	155(21.1)	1.12(0.87, 1.45)	1.11(0.85, 1.46)	
** **Q3(2.39–)	127(17.3)	0.88(0.67, 1.15)	0.90(0.68, 1.19)	
** **Q4(2.87–7.42)	93(12.7)	0.61(0.46, 0.81)	0.63(0.47, 0.86)	
** **p for trend		0.000	0.002	
** TT4RI**				0.85 (0.76, 0.95)
** **Q1(0.04–)	136(18.6)	1	1	
** **Q2(10.39–)	147(20.1)	1.10(0.85, 1.43)	1.05(.079, 1.38)	
** **Q3(19.71–)	131(17.9)	0.96(0.73, 1.25)	0.93(0.70, 1.23)	
** **Q4(31.81–122.36)	102(14.0)	0.71(0.54, 0.94)	0.72(0.54, 0.97)	
** **p for trend		0.011	0.026	
** TFQI**				0.82 (0.74, 0.91)
** **Q1(−0.86–)	161(23.1)	1	1	
** **Q2(−0.21–)	153(19.8)	0.83 (0.64, 1.06)	0.94(0.72, 1.23)	
** **Q3(0.003–)	116(14.9)	0.58 (0.45, 0.76)	0.72(0.54, 0.96)	
** **Q4(0.21–0.93)	86(12.7)	0.49 (0.36, 0.65)	0.63(0.46, 0.85)	
** **p for trend		<0.001	0.001	

Univariate and multivariable logistic regressions were used for statistical analyses with the adjusted variables including maternal age (year), education, BMI of pre-pregnancy (kg/m^2^), multi-parity (yes or no), smoking (yes or no) and alcohol drinking during early pregnancy (yes or no), family history of diabetes (yes or no). Abbreviations: GDM, gestational diabetes mellitus; FT3, free triiodothyronine; FT4, free thyroxine; TSH, thyroid-stimulating hormone; TSHI, TSH Index; TT4RI, Thyrotroph T4 Resistance Index; TFQI, Thyroid Feedback Quartile-Based index. TT4RI = FT4 (pmol/L) × TSH (mIU/L); TSHI = ln TSH (mIU/L) + 0.1345 × FT4(pmol/L); TFQI = cdf FT4 − (1 − cdf TSH). TFQI indicated the difference between FT4 quantile and the reversed TSH quantile; Cdf denoted cumulative distribution function.

Women of IH during early pregnancy had significantly increased risk of GDM (OR = 2.79; 95% CI, 1.57–4.96, *p* < 0.001) compared with women of euthyroid. No significant associations were observed in the rest of thyroid disorders, which might be due to a small sample size (**Supplementary Table S2**). Most of the characteristics of women included for analysis (n = 2,927) were comparable with those excluded (n = 644) in terms of maternal age, parity, family history of diabetes and thyroid disorders, smoking and alcohol drinking, and physical activity (**Supplementary Table S3**). The significant interactions on GDM risk were only observed between thyroid resistance indices with family history of diabetes, TFQI, and maternal age. Subgroup analyses (**Supplementary Table S4**) suggested the inverse association of central thyroid resistance indices with GDM risk was more evident among women of family history of diabetes. For TFQI, the association was more obvious in women aged above 35 years. Sensitivity analyses (data not shown) indicated that the associations were persistent when women of any thyroid dysfunction (n = 338) or any positive TAs (n = 18) were excluded.

## Discussion

### Summary of Current Findings and Implications

Our prospective data showed that both peripheral and central thyroid resistance indices during the first half of pregnancy significantly affected the subsequent risk of GDM. Higher levels of FT3 and FT3/FT4 but lower central resistance indices (TFQI, TT4RI, and TSHI) were associated with increased GDM risk or glucose intolerance, even among euthyroid women. To the best of our knowledge, this is the first study exploring the associations of central thyroid resistance indices with GDM risk and glycemic control. We identified FT3/FT4 as the proxy of peripheral thyroid sensitivity, positively associated with GDM risk. Our findings offered a new explanation for gestational thyroid profiles and implied a potentially therapeutic measure by modifying thyroid sensitivity during early pregnancy for GDM prevention.

### Results Explanations on Thyroid Function and GDM Risk

We observed that higher FT3 and FT3/FT4 were associated with increased risk of GDM or glucose intolerance. Thyroid hormones might affect GDM risk mainly through their influence on glucose intolerance. The findings are in line with prior observational studies (13, 17, 24–27) although not all (3, 16, 28, 29). Zhu and colleagues (25) reported that both first and second trimester measures of T3 and T3/FT4 were positively associated with the risk of GDM. A cross-sectional analysis (27) among 600 non-Hispanic white women also reported that fasting thyroid hormones, especially T3/fT4 ratio, were significantly associated with maternal glucose and C-peptide levels [z score sums (fasting and 1 and 2 h), *p* < 0.001], while another study in Hispanic women suggested that GDM risk increased with thyrotropin levels (28). Differences in study design, demographics of study population (i.e., race/ethnicity, iodine adequacy), and diagnostic criteria for GDM and thyroid dysfunction, even measuring time of THs during pregnancy as well, may have contributed to the divergent findings. Future studies additionally testing insulin resistance might help the results explanation. Thyroid hormones not only have an overall effect on metabolism that would prevent diabetes development by increasing glucose and fatty acid oxidation but also play a role in glucose derangement by its sensitization to catecholamines *via* increasing hepatic glycogenolysis and glucose intestinal absorption (7). T3 is the biologically active hormone responsible for stimulating endogenous glucose production and insulin secretion (30), yet its longitudinal association with GDM risk were much less studied. Our findings added new evidence to the literature that higher FT3 in the first half of pregnancy would increase GDM risk. Consistent with previous reports (3, 31, 32), our results suggested that women of higher FT4 might have lowered GDM risk. In addition, women of IH (low FT4 but normal TSH) increased almost twofold of GDM risk. An early review (33) suggested that low T4 observed in GDM mothers may be compensated by increased placental availability of T3 or T4 *via* activating TH transporters or reduced deiodinases in the feto-placental circulation. This may partly explain our findings that low FT4 or IH but high FT3/FT4 were linked with increased GDM risk. Inconsistency in previous studies implicated that TSH or THs alone may be insufficient to explain the relationship between thyroid system and glucose homeostasis.

### Results Explanation and Potential Mechanisms of Thyroid Resistance Indices on GDM Risk

Our study observed a reduced GDM risk in women of higher levels of thyroid resistance indices (both central and peripheral). The relationship remained consistent in women with euthyroid. Elevated indices of thyroid resistance has been observed to be associated with multiple unfavorable metabolic factors or disorders in general population including adipocyte fatty-acid-binding protein (34), obesity (15), type 2 diabetes, metabolic syndromes, or even diabetes-related mortality (14). However, contradictory findings were reported in others (17, 35, 36). Similar to our findings in pregnant women, several cross-sectional studies among Chinese population reported that a decreased sensitivity/increased resistance to thyroid hormones were associated with reduced risk of pre-diabetes (17), lowered levels of HbA1c (35), or body mass index (BMI) (36). A cross-sectional study (17) among Chinese adults reported that per SD increase in TSHI, TT4RI, and parametric TFQI and decrease in FT3/FT4 were associated with decreased risk of prediabetes by 9%–11%. Another community-based cross-sectional analysis (36) among 193 patients of SCH also indicated that the secretory capacity of the thyroid gland (SPINA-GT) and Jostel’s TSHI were significantly and negatively correlated with indices of obesity, suggesting low levels of thyroid homeostasis indices increased the risk of overall obesity in RTH. The discrepant findings might be related with different physiological status or severity of metabolic disorders of the studied populations. Compared with patients with diabetes (18) or morbid obesity (15), women with GDM or prediabetes had a relatively less impaired glycemic control and energy imbalance, which may present different physiological regulation on the profiles of thyroid hormones.

Several mechanisms have been proposed including the adaptive process to increase energy expenditure, the influence of leptin, changes in the activity of deiodinases, the chronic low-grade inflammation, and the presence of insulin resistance (37, 38).Gestation implies an increased burden on thyroid gland, which not only stimulates a higher production of THs but also might promote a compensation mechanism by slightly increasing TSH and THs production (within normal range) for maintenance of glucose homeostasis (39). Patients with metabolic disorders have been reported to have significantly reduced expressions of iodothyronine deiodinase (DIO) and thyroid hormone receptor (THR) in peripheral target organs (21). Several studies have shown in participants that increased circulation levels of TSH or FT4 (resistance indices to TH) were associated with improved insulin sensitivity or glucose utilization (40–42). Central thyroid sensitivity could also modify the secretion of leptin, which might affect feeding behavior or upregulate the expression of DIO1, leading to the modification of adiposity and glucose metabolism (37). The biological explanation for the possibly “protective” effect of lowered thyroid sensitivity against GDM is awaiting confirmation. Future studies further testing insulin resistance and certain cytokines and the dynamic changes in THs during gestation in the context of different severity of metabolic disorders may help explain current findings.

### Strengths and Limitations

There are several strengths in our study. First, the study had a population-based prospective design. Thyroid resistance indices offer a more systemic view and deliver important supplementary insights into the integrity of the FT4–TSH feedback relationship at pituitary level (19). Our findings extended the classical concept of separate measurements of thyroid markers and added new quantitative dimensions to evaluate thyroid homeostasis in pregnant women. Second, we have established a trimester- and assay-specific reference intervals for thyroid hormones, which allow for accurate classification of thyroid dysfunction. Third, the study was conducted in a coastal city in South China, which might reduce the potential impact of iodine deficiency for thyroid function. Moreover, we have excluded the most common factors that may influence the valid estimation of thyroid biomarkers such as assay interference from medications treatment (i.e., metformin) and application of standard assay of intracellular adhesion molecule (ICAM) for THs testing in a nationally certified laboratory (43).

Several limitations in this study should be acknowledged. First, in accordance with the clinical routines of the hospital, only pregnant women of abnormal THs were required for TAs testing. The presence of potentially unidentified thyroid autoimmunity may influence the association of thyroid markers with GDM. Second, thyroid markers were only measured during the first half of gestation. Future prospective studies assessing the dynamic changes in thyroid biomarkers cross the span of pregnancy even postpartum, and their associations with insulin sensitivity are warranted. Third, current data were from only one hospital. The findings should be confirmed in other ethnics or populations. Finally, as with other observational designs, the causal relationship might not be inferred, and potential residual confounding was unavoidable, although we have adjusted for the major confounders. The low prevalence of clinical thyroid disorders in our participants may limit the statistical power needed to find subtle associations or perform subgroup analyses. Notwithstanding, the associations were strong enough to bear statistical testing when thyroid variables were treated as continuous data.

## Conclusions

Our prospective study suggested that increased central and peripheral thyroid resistance indices during the first half of pregnancy were associated with reduced risk of GDM and glucose intolerance. Our findings added new dimensions to the evaluation of thyroid homeostasis in pregnant women. Future studies exploring the interactive impacts of thyroid variables and glucose homeostasis on obstetric outcomes may offer new insight into the linkage.

## Data Availability Statement

Some or all datasets generated and/or analyzed during the current study are not publicly available but are available from the corresponding author on reasonable request. Requests to access the datasets should be directed to ZL, liuzhm8@mail.sysu.edu.cn.

## Ethics Statement

The studies involving human participants were reviewed and approved by the Ethical Research Committee of Huizhou First Mother and Child Health-Care Hospital. The patients/participants provided their written informed consent to participate in this study.

## Author Contributions

Both ZL and GL conceptualized the topic, made results explanation, and drafted the manuscript. GL and DZ researched data and made data collection and analysis. WP coordinated the study investigation and approved data utility. GL made similar contributions to the manuscript with ZL. All authors contributed to the article and approved the submitted version.

## Funding

This work was supported by the funding of One Hundred Person Project of Sun Yat-sen University with Funding no. 51000-18841203.

## Conflict of Interest

The authors declare that the research was conducted in the absence of any commercial or financial relationships that could be construed as a potential conflict of interest.

## Publisher’s Note

All claims expressed in this article are solely those of the authors and do not necessarily represent those of their affiliated organizations, or those of the publisher, the editors and the reviewers. Any product that may be evaluated in this article, or claim that may be made by its manufacturer, is not guaranteed or endorsed by the publisher.
