# Supplementary material for: Increased Central and Peripheral Thyroid Resistance Indices During the First Half of Gestation Were Associated With Lowered Risk of Gestational Diabetes—Analyses Based on Huizhou Birth Cohort in South China
Source: Front Endocrinol (Lausanne). 2022 Mar 8;13:806256. doi: 10.3389/fendo.2022.806256 (PMC8957094; doi:10.3389/fendo.2022.806256)
Supplement: Supplementary file 1 [file DataSheet_1.docx]

**Supplemental Table A1 Diagnosis criteria of thyroid dysfunction using hospital-tailored and semester-specific cut-offs of thyroid markers**

|  | 0~12 weeks | |  | 13~20 weeks | |
| --- | --- | --- | --- | --- | --- |
|  | FT4 (pmol/L) | TSH (mIU/L) |  | FT4 (pmol/L) | TSH (mIU/L) |
| Euthyroid | 12.59~23.05 | 0.01~3.76 |  | 12.09~19.94 | 0.03~4.14 |
| Subclinical hypothyroidism | 12.59~23.05 | >3.76 |  | 12.09~19.94 | >4.14 |
| Overt hypothyroidism | <12.59 | >3.76 |  | <12.09 | >4.14 |
| Subclinical hyperthyroidism | 12.59~23.05 | <0.01 |  | 12.09~19.94 | <0.03 |
| Overt hyperthyroidism | >23.05 | <0.01 |  | >19.94 | <0.03 |
| Isolated hypothyroxinemia | <13.10 (<P_5_) | 0.01~3.76 |  | <12.43 (<P5) | 0.03~4.14 |

Total 602 eligible women who registered from January to October 2019 and conformed to the requirement of National Academy of Clinical Biochemistry (NACB) of 2017 were included to establish hospital-tailored, trimester-specific reference intervals (P_2.5_~P_97.5_) of thyroid function in the Huizhou 1^st^ Mother and Chile Health-care Hospital.

**Supplemental Table A2 Odds ratios (ORs) and 95% confident intervals (CI) for gestational diabetes mellitus (GDM) by thyroid disorders in the first-trimester**

|  | n | GDM n(%) | Crude Model | Adjusted Model* |
| --- | --- | --- | --- | --- |
| Euthyroid (reference) | 2589 | 448 (17.3) | 1 | 1 |
| Subclinical hyperthyroidism | 100 | 13 (13.0) | 0.71(0.39, 1.29) | 0.68(0.36, 1.27) |
| Overt/Clinical hyperthyroidism | 4 | 1 (25.0) | 1.59 (0.17, 15.31) | 1.56 (0.16, 15.30) |
| Subclinical hypothyroidism | 24 | 4 (16.7) | 0.95 (0.32, 2.80) | 0.81(0.26, 2.48) |
| Overt/Clinical hypothyroidism | 76 | 10 (13.2) | 0.72 (0.37, 1.42) | 0.88 (0.26, 2.48) |
| Isolated hypothyroxinemia | 55 | 24 (43.6) | 3.69(2.15, 6.39) | 2.79(1.57, 4.96) |

Multivariable logistic regression was used for statistical analysis with adjusted variables including maternal age(y), education, BMI of pre-pregnancy (kg/m^2^), multi-parity (yes or no), smoking during early pregnancy (yes or no), alcohol drinking during early pregnancy (yes or no), family history of diabetes (yes or no). The cutoffs for diagnosis of various thyroid disorders were referred to the **Supplemental Table A1**.

**Supplemental Table A3 Participants’ characteristics between women included in the analysis (n=2927) and women excluded due to thyroid markers testing later than 20 gestational weeks or unknown oral glucose tolerance testing results (n=644).**

|  | Included in the analysis (N=2927) | Excluded in the analysis (N=644) | P value |
| --- | --- | --- | --- |
| Maternal age (y) | 28.4 ±4.2 | 28.2±4.5 | 0.212 |
| Pre-pregnancy BMI (kg/m^2^) | 21.0±3.3 | 21.4±3.7 | 0.039 |
| Residential status, rural (%) | 61.1 | 62.2 | 0.847 |
| Education, college/university and above, n(%) | 27.5% | 20.6% | <0.001 |
| Multi-parity, n(%) | 45.0% | 45.8% | 0.727 |
| Medical history of GDM | 5.1% | 8.0% | 0.008 |
| 1st degree Family history of type 2 diabetes, n(%) | 8.8% | 8.7% | 0.945 |
| 1st degree Family history of thyroid disorders, n(%) | 4.9% | 6.0% | 0.280 |
| Smoking during early pregnancy, n(%) | 1.0% | 0.8% | 0.595 |
| Passive smoking | 56.4% | 56.4% | 0.984 |
| Alcohol drinking during early pregnancy, n(%) | 3.9% | 3.8% | 0.952 |
| Physical activities less than 30min/d | 35.6% | 32.3% | 0.362 |

Women included for analysis were compared with women excluded for analysis due to thyroid testing later than 20 gestational weeks (n=34) or unknown OGTT results (n=610). For continuous variables, data were presented as mean ± standard deviation and compared by student t-test. For categorical variables, data were presented as n (%) and compared by chi-square test. Smoking was defined as smoking at least once during 1^st^ trimester; alcohol drinking was defined as alcohol drinking at least once per week with more than 100 ml per time during 1^st^ trimester. Abbreviations: GDM, gestational diabetes mellitus; BMI, body mass index.

**Supplemental Table A4** Subgroup analyses by maternal age, body mass index (BMI) , parity and family history of diabetes for the association of thyroid central resistance indices by quartiles with the risk of gestational diabetes mellitus (GDM) using multivariable logistic regression models*

|  | GDM n (%) | Adjusted ORs (95% CI) | P for trend | P for interaction |
| --- | --- | --- | --- | --- |
| **TSHI** |  |  |  |  |
| Family history of DM |  |  |  | **0.085** |
| Yes |  |  |  |  |
| Q1(-3.85~ ) | 20 (33.3) | 1 |  |  |
| Q2(1.79~ ) | 20 (33.9) | 1.06(0.45, 2.47) |  |  |
| Q3(2.45~ ) | 19 (31.7) | 1.12(0.48, 2.62) |  |  |
| Q4(2.92~7.42) | 8 (13.3) | 0.31(0.12, 0.81) | 0.024 |  |
| No |  |  |  |  |
| Q1(-4.04~ ) | 120 (17.9) | 1 |  |  |
| Q2(1.74~ ) | 135 (20.1) | 1.14(0.86, 1.52) |  |  |
| Q3(2.39~ ) | 108 (16.1) | 0.91(0.68, 1.23) |  |  |
| Q4(2.86~4.46) | 86 (12.8) | 0.73(0.53, 1.00) | 0.026 |  |
| **TT4RI** |  |  |  |  |
| Family history of DM |  |  |  | **0.059** |
| Yes |  |  |  |  |
| Q1(0.04~ ) | 21 (35.0) | 1 |  |  |
| Q2(10.68~ ) | 21 (35.0) | 0.91(0.4, 2.08) |  |  |
| Q3(21.23~ ) | 16 (26.7) | 0.78(0.33, 1.87) |  |  |
| Q4(34.06~118.36) | 9 (15.3) | 0.34(0.13, 0.88) | 0.029 |  |
| No |  |  |  |  |
| Q1(0.04~ ) | 115 (17.1) | 1 |  |  |
| Q2(10.35~ ) | 124 (18.5) | 1.04(0.77, 1.4) |  |  |
| Q3(19.62~ ) | 118 (17.6) | 1.02(0.76, 1.37) |  |  |
| Q4(31.70~122.36) | 92 (13.7) | 0.78(0.57, 1.07) | 0.139 |  |
| **TFQI** |  |  |  |  |
| Age (years) |  |  |  | **<0.001** |
| <=35y |  |  |  |  |
| Q1(-0.86~ ) | 136(21.7) | 1 |  |  |
| Q2(-0.21~ ) | 140(19.3) | 0.98 (0.74, 1.29) |  |  |
| Q3(0.00~ ) | 107(14.2) | 0.73 (0.54, 0.98) |  |  |
| Q4(0.21~0.93) | 78(12.1) | 0.62 (0.45, 0.86) | 0.001 |  |
| >35y |  |  |  |  |
| Q1(-0.82~ ) | 25 (35.7) |  |  |  |
| Q2(-0.21~ ) | 13 (29.5) | 0.67 (0.28, 1.63) |  |  |
| Q3(0.00~ ) | 9 (31.0) | 0.82 (0.29, 2.31) |  |  |
| Q4(0.22~0.75) | 8 (25.8) | 0.79 (0.28, 2.21) | 0.633 |  |
| Family history of DM |  |  |  | **0.061** |
| Yes |  |  |  |  |
| Q1(-0.86~ ) | 25 (42.4) | 1 |  |  |
| Q2(-0.24~ ) | 17 (28.8) | 0.7 (0.3, 1.64) |  |  |
| Q3(0.00~ ) | 17 (27.0) | 0.65 (0.28, 1.5) |  |  |
| Q4(0.19~0.85) | 8 (13.8) | 0.26 (0.1, 0.7) | 0.006 |  |
| No |  |  |  |  |
| Q1(-0.85~ ) | 138 (21.3) | 1 |  |  |
| Q2(,0.20~ ) | 133 (19.0) | 0.96 (0.73, 1.28) |  |  |
| Q3(0.00~ ) | 99 (13.9) | 0.73 (0.54, 0.99) |  |  |
| Q4(0.19~0.93) | 79 (12.6) | 0.67 (0.49, 0.93) | 0.007 |  |

*Results were reported only for P for interaction less than 0.15 for the production term of thyroid markers with stratified variables.

Multivariable logistic regression was applied for all the analyses with adjusted variables including maternal age (y), education, smoking during early pregnancy (yes or no), and alcohol drinking during early pregnancy (yes or no), BMI of pre-pregnancy (kg/m^2^), multi-parity (yes or no) and family history of diabetes (yes or no). The stratified variable would not be controlled in the multivariable regression model if the subgroup analysis by this variable was performed. Abbreviations: OR: odds ratio; GDM: gestational diabetes mellitus; BMI: body mass index; DM: diabetes mellitus; FT3: free triiodothyronine; FT4: free thyroxine; TSH: thyroid stimulating hormone; TSHI: TSH Index; TT4RI: Thyrotroph T4 Resistance Index. TT4RI=FT4 (pmol/L)×TSH(mIU/L); TSHI=ln TSH (mIU/L) + 0.1345×FT4(pmol/L); TFQI=cdf FT4-(1-cdf TSH).TFQI indicated the difference between FT4 quantile and the reversed TSH quantile; Cdf denoted cumulative distribution function
